# Red blood cell distribution width and all-cause mortality in congestive heart failure patients: a retrospective cohort study based on the Mimic-III database

**DOI:** 10.3389/fcvm.2023.1126718

**Published:** 2023-05-03

**Authors:** Xuan Ji, Weiqi Ke

**Affiliations:** ^1^ Department of Traditional Chinese Medicine, The First Affiliated Hospital of Shantou University Medical College, Shantou, China; ^2^ Department of Anesthesiology, The First Affiliated Hospital of Shantou University Medical College, Shantou, China

**Keywords:** congestive heart failure, red blood cell distribution width, all-cause mortality, cox proportional hazards regression, RDW

## Abstract

**Background:**

The red blood cell distribution width (RDW) is a metric that measures the variation in the size of red blood cells and is presented as the red blood cell volume coefficient of variation. Increased RDW levels are closely linked to an elevated risk of death from congestive heart failure (CHF) and might be a new risk marker for cardiovascular disease. This research sought to evaluate the possible link between RDW levels and all-cause mortality in CHF patients after controlling for other covariates.

**Methods:**

The publicly accessible Mimic-III database served as the source of data for our research. We employed ICU admission scoring systems to gather information on each patient's demographical data, laboratory test results, comorbid conditions, vital signs, and scores. Among CHF patients, the link between baseline RDW levels and short-, medium-, and long-term all-cause mortality was evaluated by Cox proportional hazard analysis, smooth curve fitting, and Kaplan–Meier survival curves.

**Results:**

In total, 4,955 participants were selected for the study with an average age of 72.3 ± 13.5 years (old) and with males accounting for 53.1%. The findings recorded from the fully adjusted Cox proportional hazard model showed that higher RDW was associated with a greater risk of 30-day, 90-day, 365-day, and 4-year all-cause death; the HRs and 95% confidence intervals were 1.11 (1.05, 1.16), 1.09 (1.04, 1.13), 1.10 (1.06, 1.14), and 1.10 (1.06, 1.13), respectively. The results were stable and reliable using subgroup analysis. Smooth curve fitting and the K-M survival curve method further validated our results.

**Conclusion:**

The RDW levels had a u-shaped connection with 30-day mortality. The RDW level was linked to an elevated risk of short-, medium-, and long-term all-cause death among CHF patients.

## Introduction

End-stage heart failure is the common terminal phase of the development of most cardiovascular diseases. It is a clinical condition hallmarked by cardiac insufficiency, the activation of the neuroendocrine system, and the abnormal distribution of peripheral blood flow. The early detection and correct diagnosis of Congestive heart failure (CHF) are of great importance for the treatment and prognoses of CHF patients. At present, the diagnosis of CHF is mainly based on echocardiography and patients' chief complaints, while there is no definite index to anticipate the mortality among CHF patients.

The advantage of this measurement method over the traditional method of examining the morphology of red blood cell shape and size heterogeneity on blood smears is that it is highly accurate and objective. In recent years, it has been found that red blood cell distribution width (RDW) levels may be utilized as a biomarker of cardiovascular and cerebrovascular illnesses (HF, coronary artery disease, brain death or pulmonary arterial hypertension, etc.) (1–3), and the RDW level at admission is considerably linked to the occurrence and prognosis of CHF complications (4). Therefore, RDW performs an increasingly instrumental function in the severity and prognosis of CHF patients. For example, compared with the NYHA grading and the left ventricular ejection fraction (LVEF) (5), RDW has a more significant statistical relationship with the CHF patients' prognoses and may be used as a monitoring indicator of CHF progression (6). Previous studies on the link between RDW levels and the prognoses of patients with cardiovascular diseases have been reported (7–9), but the connection between RDW levels and short-, medium-, and long-term death among CHF patients has received less research attention (10, 11). Therefore, this research was designed to examine whether RDW levels were associated with 30-day, 90-day, 365-day, and 4-year all-cause deaths among CHF patients.

### Participants and methods

#### Study design

This study used a retrospective cohort design to investigate the relationship between RDW levels and all-cause mortality in CHF patients. The RDW level acquired at the baseline served as the independent target variable. The dependent variables encompassed the all-cause mortality in the first 30 days, 90 days, 365 days, and 4 years.

#### Study population

The Multiparameter Intelligent Monitoring in Intensive Care III Version 1.4 (Mimic-III V. 1.4) database was developed by Philips Medical, Beth Israel Deacon Medical Center, and MIT Computational Physiology Laboratory. It is a free and open public database that comprises clinical information from over 50,000 real-world patients hospitalized in the intensive care unit at Beth Israel Deacon Medical Center between 2001 and 2012 (6, 12). On the basis of International Classification of Diseases (ICD-9) codes, we successfully collected data on 8,952 individuals with congestive heart failure utilizing the PostgreSQL Structured Query Language.

#### Inclusion and exclusion criteria

The following were the requirements for participation: (1) patients with ICD-9 disease codes of 4,280–4,289 and 39,891; and (2) patients with a first admission and those with a first admission to the ICU (*n* = 8,952). The following were the exclusionary conditions: (1) patients aged <18 years; (2) patients with the length of ICU stay <24 h; (3) patients with leukemia and myelodysplastic syndrome; (4) patients with Dbsource = metavision; and (5) patients with missing baseline RDW values at ICU admission.

#### Variables

At baseline, we determine the RDW value and set it as a continuous variable in the study (13, 14). The all-cause death over 30 days, 90 days, 365 days, and 4 years was recorded as a dichotomous variable. The Social Security Death Index data were used to acquire information on survival (encompassing survival outcomes and death time).

As a result, the fully adjusted model was developed using the variables below: (1) continuous variables (obtained at baseline): age; heart rate; systolic blood pressure (SBP); temperature; pulse oxygen saturation (SPO2); diastolic blood pressure (DBP); respiratory rate; albumin level; blood urea nitrogen (Bun) level; sodium level; prothrombin time (Pt); platelet level; hemoglobin level; partial thromboplastin time (Ptt); hematocrit level; glucose level; potassium level; creatinine level; bicarbonate level; serum anion gap; red blood cell distribution width (RDW) level; red blood cell (RBC) count; white blood cells (WBC) count; the Simplified Acute Physiology Score II (SAPS II); the Sequential Organ Failure Assessment (SOFA) score; and the Elixhauser-van Walraven Comorbidity Index (EVCI); and (2) categorical variables (obtained at baseline): sex; admission type; insurance type; deficiency anemias; blood loss anemia; coagulopathy; renal failure; hypothyroidism; complicated diabetes; uncomplicated diabetes; peripheral vascular disease; hypertension; liver disease; pulmonary circulation; valvular disease; chronic pulmonary disease; and cardiac arrhythmias.

#### Statistical analysis

Continuous data are reported as the mean ± standard deviation (SD) (Gaussian distribution), and categorical data are given as percentages and frequencies. To test for differences among various RDW levels (quartiles), we employed the *χ*^2^ test (categorical data), the Kruskal–Wallis H test (skewed distribution), or one-way ANOVA (normal distribution). The link between all-cause mortality and RDW was examined by constructing three separate models utilizing multivariate and univariate Cox proportional hazards regression models, including a nonadjusted model (there were no covariates that were corrected.), a minimally adjusted model (sociodemographic characteristic was the only accounted covariate) and a fully adjusted model (adjustments were made to the factors listed in Table 1) (15). The effect sizes were calculated and the 95 percent confidence intervals were determined. Given the widespread presumption that Cox proportional hazards regression model-based approaches are incapable of dealing with nonlinear models, we utilized a Cox proportional hazards regression model incorporating cubic spline functions and smoothing curve fitting to probe into the nonlinear characteristics between RDW and all-cause death (penalized spline method). As soon as we found nonlinearity, we employed a recursive technique to compute the inflection point. Afterward, we used the two sides of the inflection point to design a two-piecewise Cox proportional hazards regression model. The Kaplan–Meier (K-M) technique was utilized to evaluate the differences in the survival rate between each subgroup of patients with RDW values at admission. A stratified Cox proportional hazards regression model was utilized to carry out the subgroup analysis. Continuous data were first transformed to categorical data depending on the clinical threshold or tertile, and thereafter we conducted interaction tests. The likelihood ratio test was executed after the tests for effect modifications of the subgroups markers were completed (16).

**Table 1:** Participant's baseline characteristics (*N* = 4955).

RDW (%) groups	Total	G1(<14)	G2 (14–15.49)	G3 (15.5–16.99)	G4(≥17)	*p*-value
Number, n	4,955	1,441	1,767	980	767	
Age (years)	72.3 ± 13.5	70.5 ± 14.2	73.7 ± 13.1	73.4 ± 13.0	71.3 ± 13.1	<0.001
Gender, *n* (%)						<0.001
Male	2,632 (53.1%)	838 (58.2%)	912 (51.6%)	484 (49.4%)	398 (51.9%)	
Female	2,323 (46.9%)	603 (41.8%)	855 (48.4%)	496 (50.6%)	369 (48.1%)	
Admission type, *n* (%)						<0.001
Emergency	3,999 (80.7%)	1,150 (79.8%)	1,391 (78.7%)	792 (80.8%)	666 (86.8%)	
Elective	783 (15.8%)	229 (15.9%)	316 (17.9%)	160 (16.3%)	78 (10.2%)	
Urgent	173 (3.5%)	62 (4.3%)	60 (3.4%)	28 (2.9%)	23 (3.0%)	
Insurance, *n* (%)						<0.001
Medicare	3,657 (73.8%)	951 (66.0%)	1,363 (77.1%)	775 (79.1%)	568 (74.1%)	
Private	980 (19.8%)	383 (26.6%)	298 (16.9%)	158 (16.1%)	141 (18.4%)	
Medicaid	236 (4.8%)	76 (5.3%)	80 (4.5%)	36 (3.7%)	44 (5.7%)	
Government	61 (1.2%)	23 (1.6%)	18 (1.0%)	9 (0.9%)	11 (1.4%)	
Self Pay	21 (0.4%)	8 (0.6%)	8 (0.5%)	2 (0.2%)	3 (0.4%)	
Vital signs						
Heart rate (bpm)	85.4 ± 15.5	85.6 ± 15.1	85.1 ± 15.1	85.3 ± 15.8	85.7 ± 16.5	0.704
SBP (mmHg)	116.9 ± 16.9	116.4 ± 16.8	117.2 ± 16.2	118.3 ± 17.4	115.0 ± 17.7	<0.001
DBP (mmHg)	56.8 ± 9.9	58.2 ± 9.7	56.8 ± 9.7	56.2 ± 9.8	55.2 ± 10.5	<0.001
Respiratory rate (bpm)	19.4 ± 4.1	19.2 ± 4.0	19.3 ± 4.1	19.5 ± 4.2	19.8 ± 4.3	0.007
Temperature (°C)	36.8 ± 0.6	37.0 ± 0.6	36.9 ± 0.6	36.8 ± 0.7	36.7 ± 0.7	<0.001
SPO_2_ (%)	97.1 ± 2.2	97.2 ± 1.8	97.1 ± 2.4	97.1 ± 2.1	97.1 ± 2.5	0.75
Laboratory parameters						
Albumin(g/dl)	3.1 ± 0.6	3.3 ± 0.5	3.1 ± 0.6	3.0 ± 0.6	3.0 ± 0.6	<0.001
Anion gap (mmol/L)	14.8 ± 3.4	14.4 ± 3.1	14.3 ± 3.3	15.1 ± 3.6	16.0 ± 4.0	<0.001
Bicarbonate (mmol/L)	24.1 ± 4.7	24.2 ± 4.3	24.2 ± 4.6	24.0 ± 5.0	23.7 ± 5.3	0.129
Creatinine (mEq/L)	1.6 ± 1.5	1.2 ± 1.0	1.5 ± 1.3	1.9 ± 1.9	2.4 ± 2.1	<0.001
Glucose (mg/dl)	150.1 ± 53.0	157.0 ± 55.8	150.5 ± 51.2	146.8 ± 51.0	140.4 ± 52.5	<0.001
Hematocrit (%)	32.2 ± 5.1	34.2 ± 5.0	32.2 ± 5.0	30.9 ± 4.5	30.2 ± 5.0	<0.001
Hemoglobin (g/dl)	10.8 ± 1.8	11.6 ± 1.8	10.8 ± 1.7	10.2 ± 1.5	9.9 ± 1.6	<0.001
Platelet (10^9^/L)	220.4 ± 105.5	224.8 ± 88.6	216.7 ± 103.6	220.3 ± 109.9	220.8 ± 130.5	0.194
Sodium(mmol/L)	138.4 ± 4.3	138.2 ± 4.1	138.5 ± 4.2	138.8 ± 4.3	138.1 ± 5.0	0.002
Potassium (mmol/L)	4.3 ± 0.6	4.2 ± 0.5	4.3 ± 0.5	4.3 ± 0.6	4.3 ± 0.6	<0.001
Ptt (seconds)	42.1 ± 21.3	43.2 ± 23.0	42.4 ± 21.3	40.1 ± 19.6	42.0 ± 20.2	0.008
Pt (seconds)	15.9 ± 5.7	14.8 ± 3.5	15.8 ± 5.3	16.4 ± 5.6	17.8 ± 8.8	<0.001
Bun (mg/dl)	32.6 ± 23.1	25.2 ± 17.4	30.9 ± 21.2	37.7 ± 24.8	44.0 ± 28.2	<0.001
WBC (10^9^/L)	12.8 ± 10.3	12.9 ± 5.1	12.5 ± 6.5	12.8 ± 8.5	13.2 ± 21.2	<0.001
RDW (%)	15.2 ± 1.9	13.3 ± 0.5	14.7 ± 0.4	16.1 ± 0.4	18.7 ± 1.7	<0.001
RBC (10^12^/L)	3.6 ± 0.6	3.8 ± 0.6	3.6 ± 0.6	3.5 ± 0.6	3.4 ± 0.7	<0.001
Scoring systems						
SOFA	4.9 ± 2.9	4.2 ± 2.6	4.8 ± 2.8	5.2 ± 3.0	5.8 ± 3.2	<0.001
SAPSII	39.7 ± 12.9	36.6 ± 12.2	39.6 ± 12.5	41.5 ± 12.8	43.8 ± 14.0	<0.001
EVCI	8.0 ± 7.0	5.7 ± 6.2	7.4 ± 6.5	9.8 ± 7.1	11.5 ± 7.6	<0.001
Comorbidities, *n* (%)						
Cardiac arrhythmias	1,177 (23.8%)	234 (16.2%)	413 (23.4%)	290 (29.6%)	240 (31.3%)	<0.001
Valvular disease	464 (9.4%)	94 (6.5%)	169 (9.6%)	109 (11.1%)	92 (12.0%)	<0.001
Pulmonary circulation	184 (3.7%)	25 (1.7%)	61 (3.5%)	54 (5.5%)	44 (5.7%)	<0.001
Peripheral vascular	555 (11.2%)	112 (7.8%)	223 (12.6%)	131 (13.4%)	89 (11.6%)	<0.001
Hypertension	672 (13.6%)	89 (6.2%)	225 (12.7%)	195 (19.9%)	163 (21.3%)	<0.001
Chronic pulmonary	1,202 (24.3%)	287 (19.9%)	441 (25.0%)	266 (27.1%)	208 (27.1%)	<0.001
Diabetes uncomplicated	1,199 (24.2%)	334 (23.2%)	444 (25.1%)	246 (25.1%)	175 (22.8%)	0.41
Diabetes complicated	440 (8.9%)	74 (5.1%)	150 (8.5%)	117 (11.9%)	99 (12.9%)	<0.001
Hypothyroidism	460 (9.3%)	94 (6.5%)	168 (9.5%)	118 (12.0%)	80 (10.4%)	<0.001
Renal failure	876 (17.7%)	104 (7.2%)	273 (15.4%)	260 (26.5%)	239 (31.2%)	<0.001
Liver disease	173 (3.5%)	17 (1.2%)	36 (2.0%)	51 (5.2%)	69 (9.0%)	<0.001
Coagulopathy	558 (11.3%)	84 (5.8%)	181 (10.2%)	134 (13.7%)	159 (20.7%)	<0.001
Blood loss anemia	120 (2.4%)	18 (1.2%)	37 (2.1%)	31 (3.2%)	34 (4.4%)	<0.001
Deficiency anemias	897 (18.1%)	186 (12.9%)	317 (17.9%)	203 (20.7%)	191 (24.9%)	<0.001
30-day mortality, *n* (%)	935 (18.9%)	192 (13.3%)	268 (15.2%)	234 (23.9%)	241 (31.4%)	<0.001
90-day mortality, *n* (%)	1,324 (26.7%)	258 (17.9%)	396 (22.4%)	333 (34.0%)	337 (43.9%)	<0.001
365-day mortality, *n* (%)	1,897 (38.3%)	376 (26.1%)	595 (33.7%)	451 (46.0%)	475 (61.9%)	<0.001
4-year mortality, *n* (%)	2,761 (55.7%)	569 (39.5%)	931 (52.7%)	660 (67.3%)	601 (78.4%)	<0.001

SBP, Systolic blood pressure; RBC, red blood cell; PTT partial thromboplastin time; BUN, blood urea nitrogen; WBC, white blood cell; PT, prothrombin time; RDW, Red Blood Cell Distribution Width; DBP, Diastolic blood pressure; SAPSII, simplified acute physiology score II; EVCI, Elixhauser-van Walraven Comorbidity Index; SOFA, sequential organ failure assessment.

A sensitivity analysis was undertaken to validate our findings' robustness. We transformed RDW into a categorical variable predicated on the clinical cutoff value and computed the P for the pattern to validate the findings obtained when RDW was used as a continuous variable and to investigate the potential of nonlinearity in the distribution.

Statistical testing was accomplished with the help of the statistical software program R (http://www.R-project.org, The R Foundation) and EmpowerStats (http://www. empowerstats.com, X&Y Solutions, Inc., Boston, MA); The statistically significant differences were identified at *p* < 0.05 (17).

## Results

### Baseline characteristics

**Figure 1:**
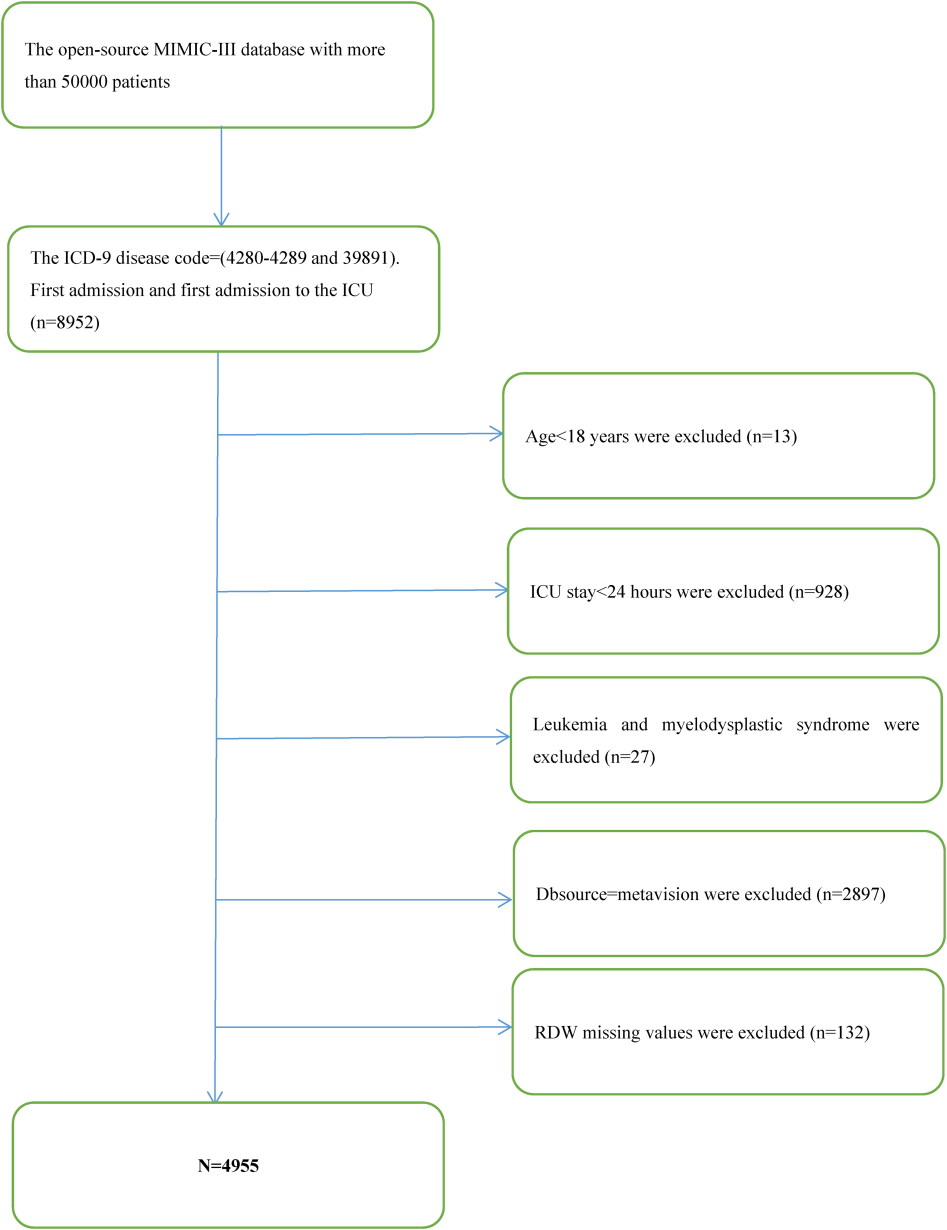
Flowchart of patient selection.

After filtering by eligibility requirements, 4,955 individuals were selected for the final analysis of data (see the flow chart in Figure 1). Table 1 summarizes the baseline features of these selected participants according to the clinical cutoff points of the RDW (%) groups. Males constituted around 53.1 percent of the 4,955 individuals that were selected, with an average age of 72.3 ± 13.5 years. The findings illustrated no statistically significant difference in the heart rate, SPO2, bicarbonate level, platelet level, or uncomplicated diabetes across the distinct RDW (%) groups (all *p* values >0.05). The subjects within the highest RDW (%) group (RDW ≥ 17%) had elevated values for the respiratory rate, serum anion gap, creatinine, sodium, potassium, Pt, Bun, WBC count, SOFA score, SAPS II score, and EVCI score and consisted of more patients with emergencies, coagulopathy, complicated diabetes, hypertension, pulmonary circulation, renal failure, valvular disease, liver disease, cardiac arrhythmias, blood loss anemia, and deficiency anemias in contrast with those in the other subgroups. Opposite trends were detected for SBP, DBP, temperature, albumin level, bicarbonate level, glucose level, hematocrit level, hemoglobin level, RBC count, and complicated diabetes.

### Results of the adjusted and unadjusted Cox proportional hazard models

As part of this investigation, we developed 3 models to evaluate the independent impact of RDW levels on all-cause death (multivariate and univariate Cox proportional hazard models). Table 2 depicts the effect sizes [hazard ratios (HRs)] and 95 percent confidence intervals. As previously stated, the model-based effect size may be defined by the difference between the unit of RDW linked to the risk of mortality in the unadjusted model (Model 1). The effect size of 1.19 for 365-day all-cause death in the unadjusted model implies that the unit difference in RDW is related to a 19-percentage increase in the mortality risk. There was a 19% increase in mortality risk in the minimally-adjusted model (Model 2) due to a change in the RDW unit. A difference in the RDW unit for the fully adjusted model (Model 3) (adjustments were made for all variables displayed in Table 1), was linked to an elevated mortality risk of 10%. To carry out sensitivity analysis, the RDW level from continuous data was transformed into categorical data (clinical cut point for RDW), and the P in the fully adjusted model for the RDW level pattern as a categorical variable was similar to the findings obtained from the RDW level as a continuous variable. Furthermore, we discovered that the effect size pattern in various RDW subgroups was non-equidistant. The findings for all-cause death over 30 days, 90 days, and 4 years were congruent to those of 365-days, which were stable and reliable. Table 2 presents the findings.

**Table 2:** Association of RDW with mortality.

Variable	Crude model HR (95% CIs) *P*-value	Model I HR (95% CIs) *p*-value	Model II HR (95% CIs) *p*-value
30-day mortality, *n* (%)			
RDW (%)	1.17 (1.14, 1.20) <0.0001	1.17 (1.14, 1.20) <0.0001	1.11 (1.05, 1.16) <0.0001
RDW (%) groups			
<14	Reference	Reference	Reference
≥14, <15.5	1.15 (0.96, 1.38) 0.1379	1.08 (0.90, 1.30) 0.4138	0.83 (0.61, 1.11) 0.2109
≥15.5, <17	1.88 (1.56, 2.28) <0.0001	1.79 (1.48, 2.17) <0.0001	1.26 (0.90, 1.74) 0.1743
≥17	2.63 (2.17, 3.17) <0.0001	2.55 (2.11, 3.09) <0.0001	1.39 (0.99, 1.96) 0.0605
90-day mortality, *n* (%)			
RDW (%)	1.18 (1.15, 1.21) <0.0001	1.18 (1.16, 1.21) <0.0001	1.09 (1.04, 1.13) 0.0002
RDW (%) groups			
<14	Reference	Reference	Reference
≥14, <15.5	1.28 (1.09, 1.49) 0.0023	1.19 (1.02, 1.40) 0.0278	0.86 (0.67, 1.12) 0.2586
≥15.5, <17	2.06 (1.75, 2.43) <0.0001	1.96 (1.66, 2.31) <0.0001	1.28 (0.96, 1.70) 0.0870
≥17	2.89 (2.46, 3.40) <0.0001	2.83 (2.40, 3.33) <0.0001	1.36 (1.01, 1.83) 0.0446
365-day mortality, *n* (%)			
RDW (%)	1.19 (1.17, 1.21) <0.0001	1.19 (1.17, 1.22) <0.0001	1.10 (1.06, 1.14) <0.0001
RDW (%) groups			
<14	Reference	Reference	Reference
≥14, <15.5	1.34 (1.18, 1.53) <0.0001	1.25 (1.10, 1.42) 0.0008	0.92 (0.74, 1.15) 0.4572
≥15.5, <17	2.03 (1.77, 2.32) <0.0001	1.91 (1.66, 2.19) <0.0001	1.33 (1.04, 1.69) 0.0232
≥17	3.11 (2.72, 3.56) <0.0001	3.06 (2.67, 3.50) <0.0001	1.52 (1.18, 1.97) 0.0012
4-year mortality, *n* (%)			
RDW (%)	1.19 (1.17, 1.21) <0.0001	1.19 (1.17, 1.21) <0.0001	1.10 (1.06, 1.13) <0.0001
RDW (%) groups			
<14	Reference	Reference	Reference
≥14, <15.5	1.45 (1.31, 1.61) <0.0001	1.34 (1.21, 1.49) <0.0001	0.99 (0.83, 1.19) 0.9351
≥15.5, <17	2.18 (1.94, 2.43) <0.0001	2.03 (1.82, 2.28) <0.0001	1.41 (1.15, 1.74) 0.0009
≥17	3.10 (2.76, 3.48) <0.0001	3.06 (2.73, 3.44) <0.0001	1.63 (1.31, 2.03) <0.0001

RDW Red Blood Cell Distribution Width; HR Hazard Ratio.

#### The results of nonlinearity of RWD and all-cause mortality

**Figure 2:**
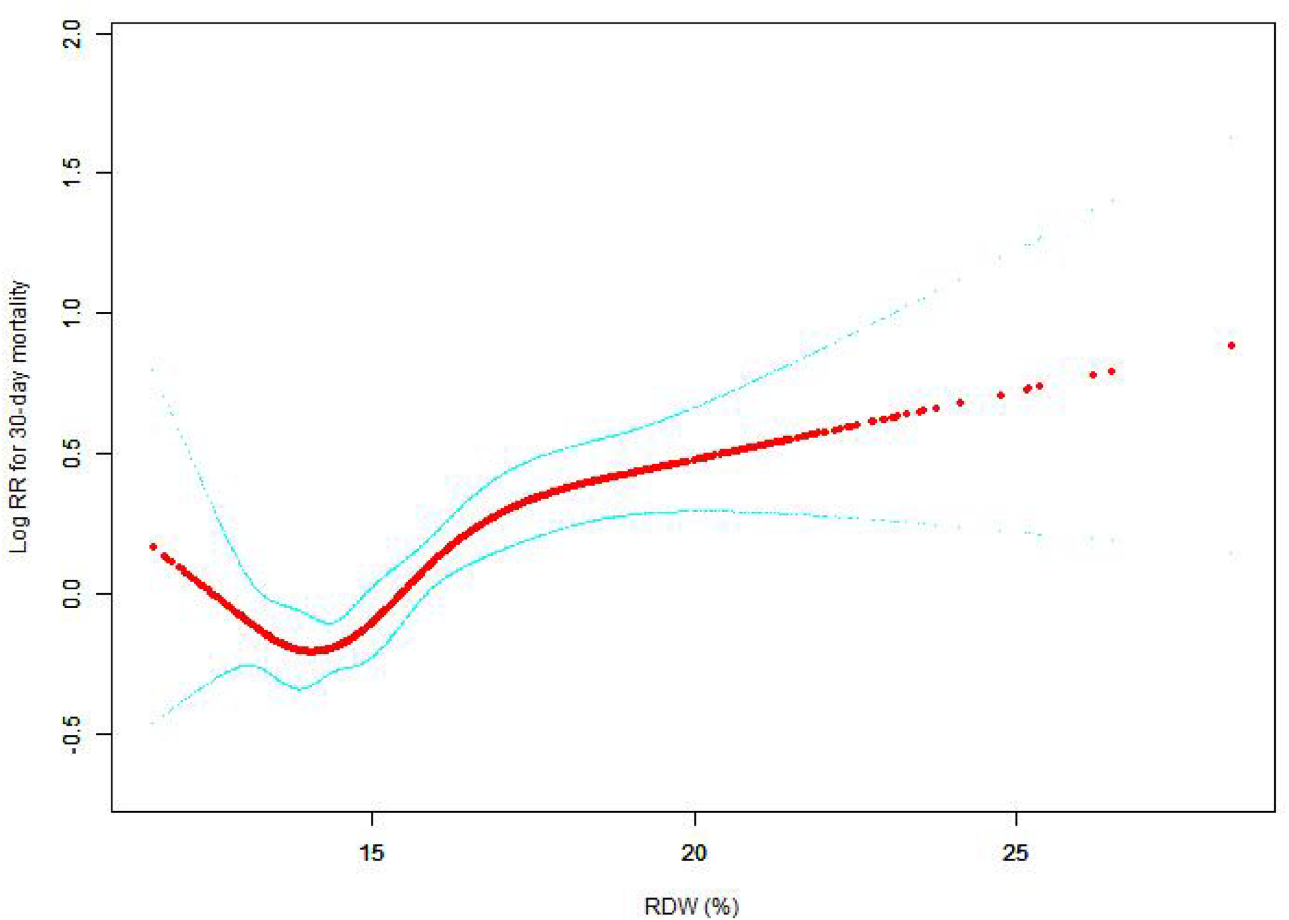
Association between RDW and 30-day all-cause mortality. A generalized additive model (GAM) revealed a threshold, nonlinear relationship between RDW and 30-day all-cause death. The smooth curve fit between variables is shown by a solid red line. The 95 percent confidence interval from the fit is represented by imaginary lines.

**Figure 3:**
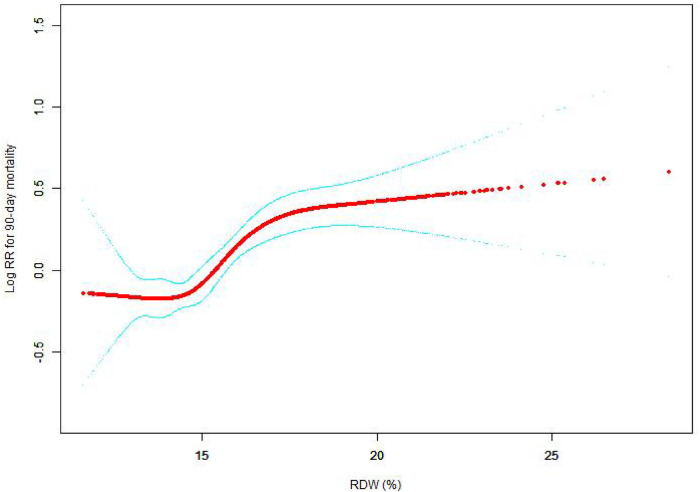
Association between RDW and 90-day all-cause mortality. A generalized additive model (GAM) revealed a threshold, nonlinear relationship between RDW and 90-day all-cause death. The smooth curve fit between variables is shown by a solid red line. The 95 percent confidence interval from the fit is represented by imaginary lines.

**Figure 4:**
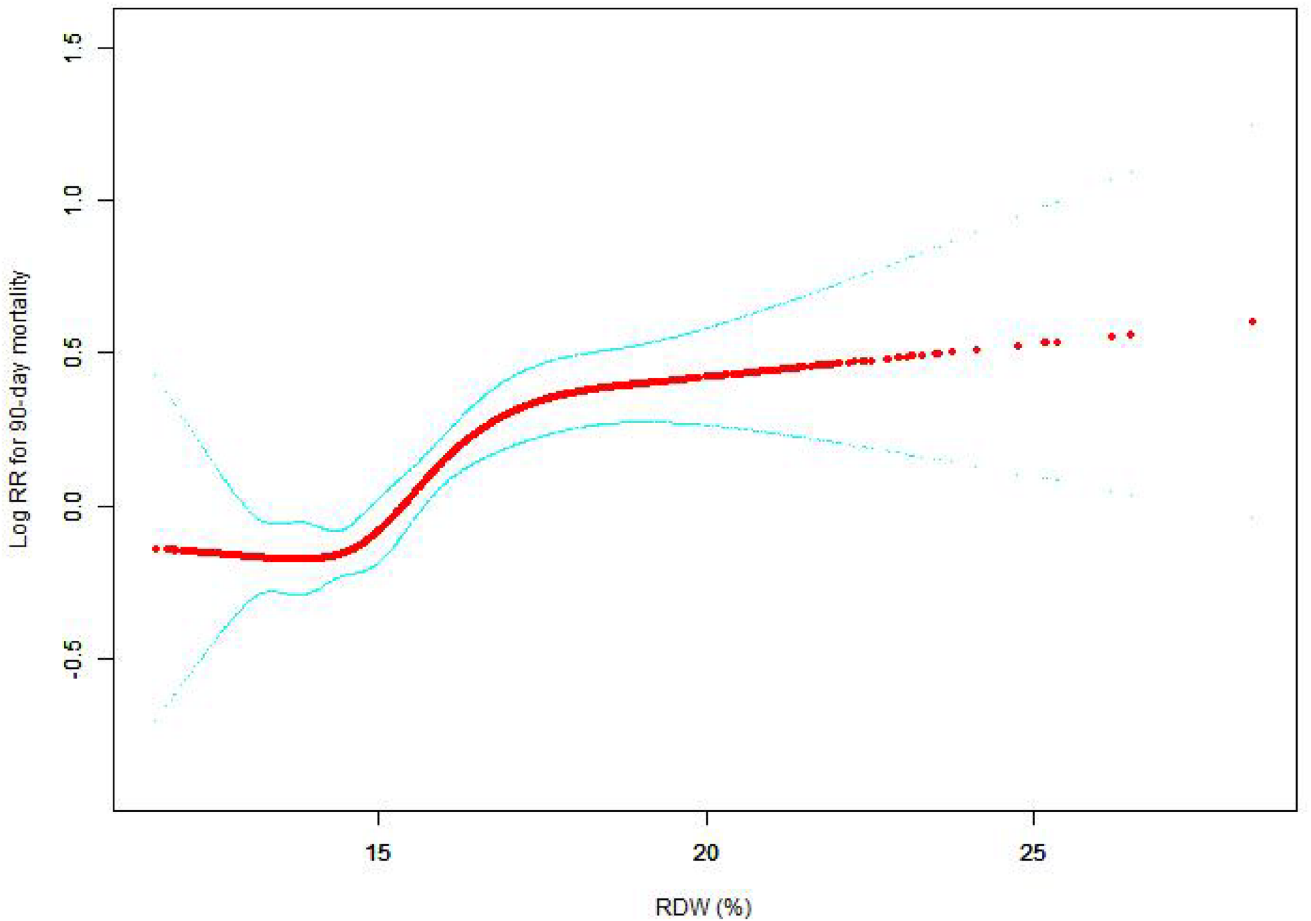
Association between RDW and 365-day all-cause mortality. A generalized additive model (GAM) revealed a threshold, nonlinear relationship between RDW and 365-day all-cause death. The smooth curve fit between variables is shown by a solid red line. The 95 percent confidence interval from the fit is represented by imaginary lines.

**Figure 5:**
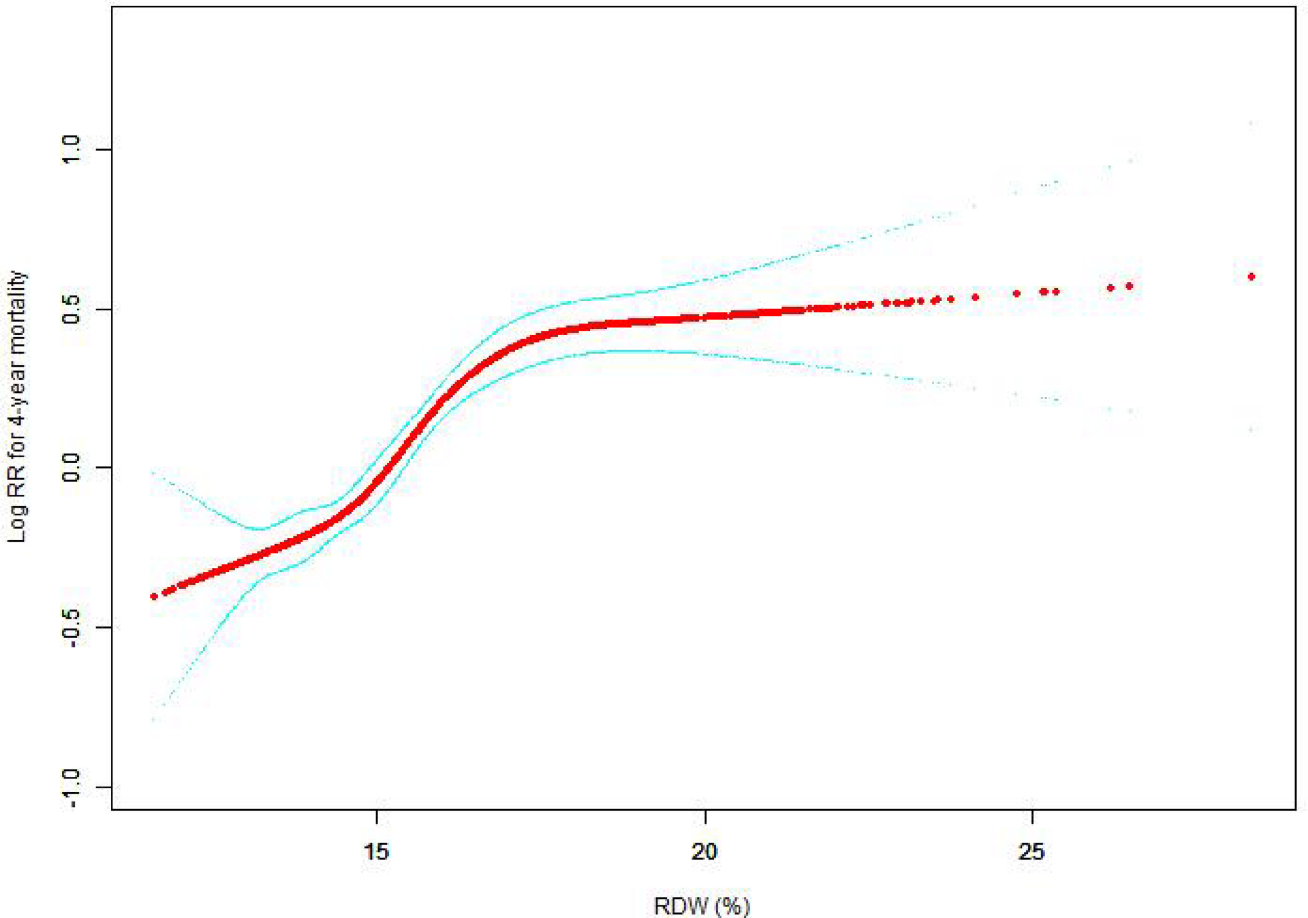
Association between RDW and 4-year all-cause mortality. A generalized additive model (GAM) revealed a threshold, nonlinear relationship between RDW and 4-year all-cause death. The smooth curve fit between variables is shown by a solid red line. The 95 percent confidence interval from the fit is represented by imaginary lines.

The nonlinear connection between RDW levels and all-cause death was investigated in the current research (Figures 2–5). The smooth curve fitting and the findings reported from the Cox proportional hazards regression model integrating cubic spline functions revealed a nonlinear link between RDW levels following the adjustment of the covariates below: age; potassium level; DBP; platelet level; temperature; hematocrit level; albumin level; creatinine level; bicarbonate level; serum anion gap; SPO2; glucose level; respiratory rate; hemoglobin level; SBP; sodium level; heart rate; Ptt; Pt; Bun level; WBC count; RDW level; RBC count; SOFA score; SAPS II score; EVCI score; sex; admission type; insurance type; deficiency anemias; peripheral vascular disease; coagulopathy; complicated diabetes; uncomplicated diabetes; hypothyroidism; chronic pulmonary disease; renal failure; hypertension; liver disease; pulmonary circulation; blood loss anemia; valvular disease; and cardiac arrhythmias. To fit the relationship, we utilized the two-piecewise Cox proportional hazard model and the Cox proportional hazard model. Based on the *p*-value obtained from the log-likelihood ratio test, we determined the best-suited model. We found a U-shaped relationship between RDW values and 30-day mortality in our study population (as shown in Figure 2); a positive correlation between RDW and 90-day mortality for RDW values higher than 15.5% (as shown in Figure 3); and a significant positive correlation between RDW and 365-day and 4-year mortality, with higher values of RDW as mortality also increased (as shown in Figures 4, 5). What's more, the largest difference in all-cause mortality between the groups occurred in the first few days of follow-up. Over time, the curves for each group became more parallel.

#### Survival status of the patients with different admission RDW levels

**Figure 6:**
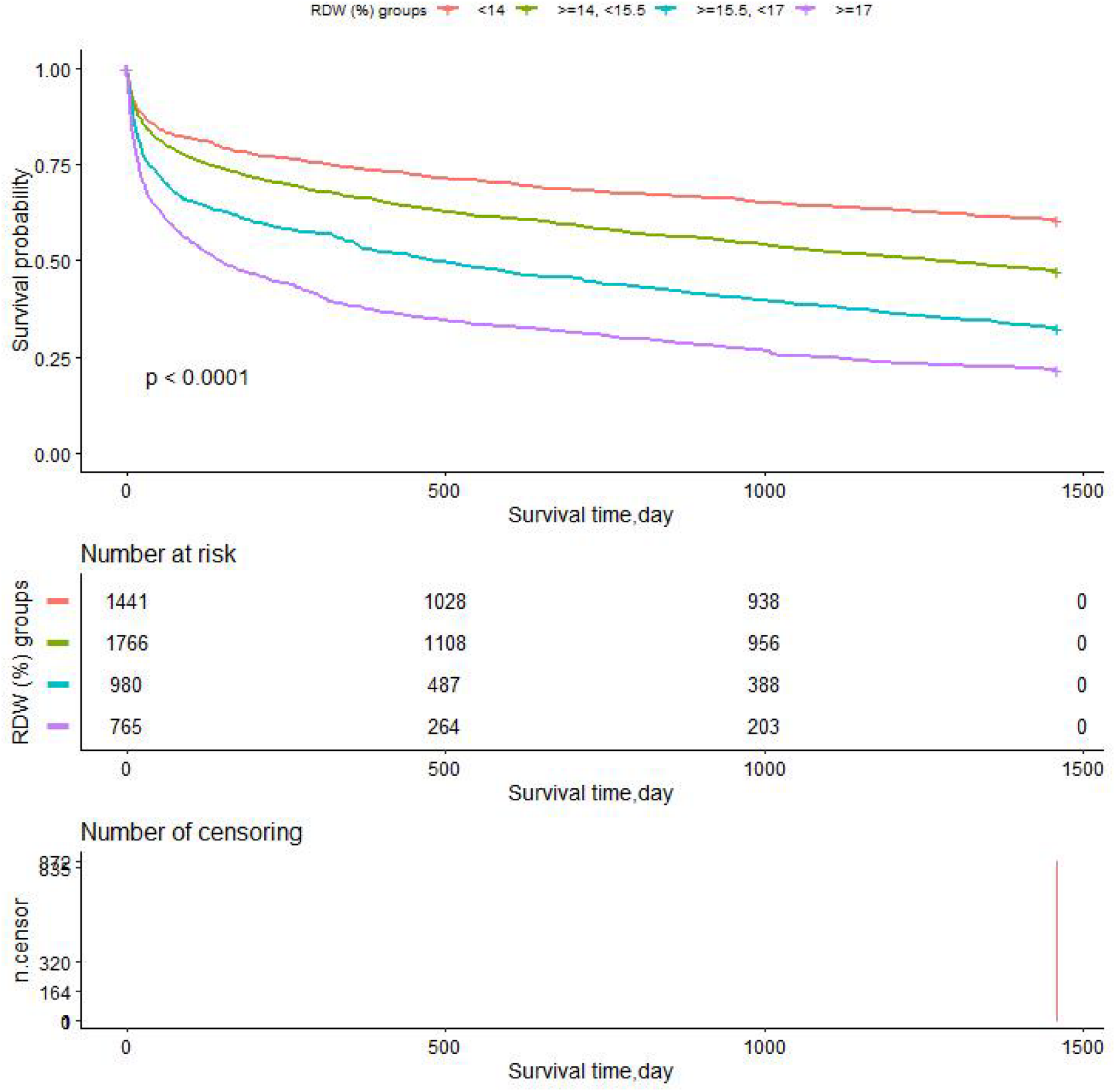
Kaplan-Meier survival curves demonstrating differences in overall survival (years).

We performed Kaplan-Meier survival curves based on RDW groups, with all-cause mortality as the dependent variable for the primary outcome indicator. The K-M survival curve illustrated that the patients' survival time values in each RDW group were G1 > G2 > G3 > G4 at any time within the 4 years (*p *< 0.0001), as shown in Figure 6. Survival probability of the CHF patients decreases as time increases, and moreover, we found that the group with RDW higher than 17 (G4) group showed the lowest survival probability.

#### Subgroup analysis

We used age (years), sex, admission type, insurance type, SBP (mmHg), respiratory rate (bpm), SPO2 (%), DBP (mmHg), heart rate (bpm), albumin level (mmol/dl), temperature (°C), bicarbonate level (mmol/L), WBC count (10^9^/L), sodium level (mmol/L), platelet level (10^9^/L), creatinine level (mEq/L), potassium level (mmol/L), serum anion gap (mmol/L), Ptt (seconds), Pt (seconds), hematocrit level (%), Bun level (mg/dl), hemoglobin level (g/dl), RBC count (10^12^/L), glucose level (mg/dl), SOFA score, SAPS II score, EVCI score, blood loss anemia, uncomplicated diabetes, chronic pulmonary disease, complicated diabetes, hypertension, hypothyroidism, peripheral vascular disease, renal failure, pulmonary circulation, liver disease, valvular disease, coagulopathy, cardiac arrhythmias, and deficiency anemias as the stratification parameters to examine the patterns of their effect sizes (Table 3). Depending on *a priori* specification, only a relatively small number of interactions were discovered.

**Table 3:** Subgroup's RWD effect size on mortality in prespecified and exploratory subgroups.

Characteristic		30-day mortality, *n* (%)		90-day mortality, *n* (%)		365-day mortality, *n* (%)		4-year mortality, *n* (%)	
	*N*	HR (95% CI)	*p* for interaction	HR (95% CI)	*p* for interaction	HR (95% CI)	*p* for interaction	HR (95% CI)	*p* for interaction
Age (years) group			0.0243		0.0003		<0.0001		<0.0001
<60	897	1.27 (1.17, 1.37)		1.31 (1.23, 1.40)		1.35 (1.27, 1.42)		1.34 (1.28, 1.40)	
≥60, <80	2,359	1.20 (1.15, 1.24)		1.20 (1.16, 1.24)		1.21 (1.18, 1.24)		1.21 (1.18, 1.24)	
≥80	1,699	1.13 (1.08, 1.18)		1.13 (1.09, 1.17)		1.14 (1.10, 1.17)		1.12 (1.09, 1.15)	
Gender			0.5648		0.7599		0.0630		0.0008
Male	2,632	1.17 (1.13, 1.21)		1.18 (1.15, 1.22)		1.21 (1.18, 1.24)		1.22 (1.19, 1.25)	
Female	2,323	1.18 (1.14, 1.23)		1.18 (1.14, 1.22)		1.17 (1.13, 1.20)		1.15 (1.12, 1.18)	
Admission type			0.1415		0.1651		0.0335		0.0088
Emergency	3,999	1.16 (1.12, 1.19)		1.17 (1.14, 1.19)		1.18 (1.15, 1.20)		1.17 (1.15, 1.19)	
Elective	783	1.25 (1.12, 1.40)		1.25 (1.15, 1.37)		1.24 (1.16, 1.33)		1.26 (1.20, 1.33)	
Urgent	173	1.30 (1.13, 1.49)		1.26 (1.11, 1.42)		1.32 (1.19, 1.47)		1.31 (1.19, 1.44)	
Insurance			0.1690		0.0958		0.0119		0.0001
Medicare	3,657	1.17 (1.13, 1.20)		1.17 (1.14, 1.20)		1.17 (1.15, 1.20)		1.17 (1.15, 1.19)	
Private	980	1.23 (1.16, 1.31)		1.25 (1.19, 1.32)		1.27 (1.22, 1.33)		1.28 (1.24, 1.33)	
Medicaid	236	1.05 (0.89, 1.24)		1.11 (0.97, 1.26)		1.21 (1.10, 1.33)		1.16 (1.07, 1.26)	
Government	61	1.06 (0.79, 1.43)		1.15 (0.94, 1.40)		1.18 (1.00, 1.40)		1.12 (0.95, 1.32)	
Self Pay	21	0.88 (0.56, 1.39)		0.88 (0.56, 1.39)		0.88 (0.56, 1.39)		0.92 (0.61, 1.38)	
Heart rate (bpm)			0.6501		0.6136		0.3356		0.9243
≥60, <90	3,018	1.18 (1.14, 1.23)		1.19 (1.15, 1.22)		1.20 (1.17, 1.23)		1.19 (1.17, 1.22)	
<60	175	1.20 (1.00, 1.43)		1.22 (1.05, 1.42)		1.29 (1.14, 1.45)		1.23 (1.11, 1.36)	
≥90	1,726	1.15 (1.11, 1.20)		1.16 (1.12, 1.20)		1.17 (1.14, 1.21)		1.18 (1.15, 1.21)	
SBP (mmHg)			0.0107		0.0011		0.0399		0.0318
≥90, <140	4,306	1.19 (1.15, 1.22)		1.20 (1.17, 1.22)		1.20 (1.18, 1.22)		1.20 (1.18, 1.22)	
<90	109	1.06 (0.94, 1.19)		1.08 (0.98, 1.20)		1.13 (1.03, 1.24)		1.12 (1.03, 1.23)	
≥140	504	1.04 (0.94, 1.15)		1.03 (0.94, 1.13)		1.10 (1.02, 1.18)		1.11 (1.05, 1.18)	
DBP (mmHg)			0.0418		0.0324		0.0966		0.2677
≥60, <90	1,626	1.14 (1.08, 1.20)		1.15 (1.10, 1.20)		1.16 (1.11, 1.20)		1.16 (1.12, 1.20)	
<60	3,270	1.18 (1.14, 1.22)		1.19 (1.16, 1.22)		1.20 (1.18, 1.23)		1.20 (1.18, 1.22)	
≥90	23	0.42 (0.12, 1.45)		0.42 (0.12, 1.45)		0.82 (0.45, 1.49)		1.18 (0.81, 1.72)	
Respiratory rate(bpm)			0.6622		0.8477		0.5847		0.3105
≥12, <20	2,918	1.18 (1.13, 1.22)		1.18 (1.15, 1.22)		1.20 (1.17, 1.23)		1.21 (1.18, 1.23)	
<12	59	1.05 (0.77, 1.42)		1.17 (0.94, 1.47)		1.22 (1.00, 1.47)		1.17 (0.99, 1.38)	
≥20	1,937	1.16 (1.12, 1.20)		1.17 (1.13, 1.21)		1.17 (1.14, 1.21)		1.17 (1.14, 1.19)	
Temperature (°C)			0.2091		0.7939		0.5387		0.6911
≥36.3, <37.2	2,739	1.17 (1.13, 1.22)		1.18 (1.14, 1.21)		1.19 (1.16, 1.23)		1.19 (1.17, 1.22)	
<36.3	866	1.18 (1.13, 1.24)		1.17 (1.13, 1.22)		1.18 (1.14, 1.23)		1.18 (1.14, 1.22)	
≥37.2	1,310	1.11 (1.05, 1.18)		1.15 (1.10, 1.21)		1.16 (1.11, 1.21)		1.16 (1.13, 1.20)	
SPO_2_ (%)			0.3678		0.3122		0.2262		0.3505
≥95	4,315	1.17 (1.14, 1.21)		1.18 (1.16, 1.21)		1.20 (1.17, 1.22)		1.19 (1.17, 1.21)	
<95	602	1.14 (1.07, 1.21)		1.15 (1.09, 1.21)		1.15 (1.10, 1.21)		1.16 (1.12, 1.21)	
Albumin(g/dl)			0.0180		0.0740		0.0310		0.2105
≥3.5	606	1.04 (0.95, 1.14)		1.08 (1.00, 1.17)		1.10 (1.03, 1.17)		1.13 (1.07, 1.19)	
<3.5	1,350	1.17 (1.13, 1.22)		1.17 (1.13, 1.21)		1.18 (1.15, 1.22)		1.17 (1.14, 1.20)	
Anion gap (mmol/L)			0.0553		0.0053		0.0037		0.0021
≥8, <16	3,113	1.19 (1.15, 1.24)		1.20 (1.17, 1.24)		1.21 (1.18, 1.24)		1.21 (1.19, 1.24)	
<8	35	1.72 (0.52, 5.72)		2.02 (0.91, 4.52)		1.71 (0.97, 2.99)		1.62 (1.05, 2.52)	
≥16	1,661	1.12 (1.07, 1.16)		1.12 (1.08, 1.16)		1.14 (1.10, 1.17)		1.13 (1.10, 1.16)	
Bicarbonate (mmol/L)			0.2299		0.1526		0.0718		0.0098
≥22, <27	2,295	1.19 (1.14, 1.24)		1.20 (1.16, 1.24)		1.22 (1.19, 1.25)		1.22 (1.19, 1.25)	
<22	1,462	1.17 (1.12, 1.22)		1.17 (1.13, 1.22)		1.17 (1.13, 1.21)		1.16 (1.13, 1.20)	
≥27	1,187	1.12 (1.06, 1.19)		1.14 (1.09, 1.19)		1.16 (1.12, 1.20)		1.16 (1.12, 1.19)	
Creatinine (mEq/L)			0.0769		0.0685		0.0770		0.0170
≥0.5, <1.2	2,458	1.19 (1.14, 1.24)		1.19 (1.15, 1.23)		1.19 (1.16, 1.23)		1.20 (1.17, 1.23)	
<0.5	108	1.26 (1.03, 1.54)		1.30 (1.09, 1.54)		1.32 (1.15, 1.53)		1.24 (1.09, 1.41)	
≥1.2	2,388	1.12 (1.08, 1.16)		1.13 (1.10, 1.17)		1.15 (1.12, 1.18)		1.14 (1.12, 1.17)	
Glucose (mg/dl)			0.1137		0.3204		0.6132		0.5042
≥70, <110	789	1.25 (1.18, 1.32)		1.22 (1.16, 1.29)		1.21 (1.16, 1.26)		1.19 (1.15, 1.23)	
<70	27	1.12 (0.90, 1.40)		1.24 (1.04, 1.47)		1.26 (1.07, 1.49)		1.30 (1.11, 1.52)	
≥110	4,136	1.16 (1.12, 1.19)		1.17 (1.14, 1.20)		1.18 (1.16, 1.21)		1.18 (1.16, 1.21)	
Hematocrit (%)			0.1365		0.0548		0.0674		0.1339
≥37, <50	863	1.21 (1.12, 1.32)		1.24 (1.16, 1.33)		1.23 (1.16, 1.31)		1.24 (1.18, 1.30)	
<37	4,079	1.17 (1.14, 1.21)		1.17 (1.15, 1.20)		1.19 (1.16, 1.21)		1.18 (1.16, 1.20)	
≥50	13	0.43 (0.11, 1.64)		0.43 (0.11, 1.64)		0.43 (0.11, 1.64)		1.04 (0.72, 1.51)	
Hemoglobin (g/dl)			0.1313		0.0265		0.0137		0.0030
≥11, <16.5	1,997	1.23 (1.17, 1.30)		1.24 (1.19, 1.30)		1.25 (1.20, 1.30)		1.25 (1.21, 1.29)	
<11	2,937	1.17 (1.13, 1.21)		1.17 (1.14, 1.20)		1.18 (1.15, 1.21)		1.17 (1.15, 1.20)	
≥16.5	21	0.74 (0.30, 1.83)		0.74 (0.30, 1.83)		0.84 (0.42, 1.67)		1.12 (0.83, 1.51)	
Platelet (10^9^/L)			0.5767		0.1179		0.0525		0.0233
≥100, <300	3,739	1.17 (1.13, 1.21)		1.18 (1.15, 1.21)		1.20 (1.17, 1.22)		1.20 (1.18, 1.22)	
<100	357	1.21 (1.12, 1.30)		1.22 (1.14, 1.30)		1.21 (1.14, 1.28)		1.18 (1.12, 1.24)	
≥300	858	1.14 (1.08, 1.21)		1.13 (1.07, 1.18)		1.14 (1.09, 1.18)		1.14 (1.10, 1.18)	
Sodium (mmol/L)			0.4504		0.3896		0.2248		.0.1554
≥135, <145	3,873	1.15 (1.12, 1.19)		1.17 (1.14, 1.21)		1.19 (1.16, 1.22)		1.19 (1.17, 1.21)	
<135	805	1.19 (1.14, 1.25)		1.19 (1.14, 1.24)		1.19 (1.15, 1.23)		1.18 (1.14, 1.22)	
≥145	276	1.14 (1.05, 1.23)		1.12 (1.04, 1.20)		1.12 (1.04, 1.19)		1.12 (1.05, 1.19)	
Potassium (mmol/L)			0.6283		0.4602		0.3379		0.0501
≥3.5, <5.5	4,561	1.17 (1.14, 1.21)		1.18 (1.16, 1.21)		1.20 (1.17, 1.22)		1.20 (1.18, 1.22)	
<3.5	248	1.19 (1.09, 1.30)		1.18 (1.10, 1.27)		1.16 (1.08, 1.24)		1.13 (1.07, 1.20)	
≥5.5	146	1.09 (0.94, 1.28)		1.09 (0.96, 1.24)		1.13 (1.02, 1.24)		1.11 (1.02, 1.22)	
Ptt (seconds)			0.1222		0.1946		0.1582		0.0961
≥31, <43	1,550	1.22 (1.16, 1.28)		1.21 (1.17, 1.26)		1.22 (1.18, 1.26)		1.22 (1.19, 1.26)	
<31	1,641	1.17 (1.11, 1.23)		1.17 (1.12, 1.22)		1.17 (1.13, 1.22)		1.19 (1.15, 1.22)	
≥43	1,380	1.13 (1.08, 1.19)		1.15 (1.11, 1.20)		1.17 (1.13, 1.20)		1.16 (1.13, 1.20)	
Pt (seconds)			0.1456		0.2829		0.1604		0.0432
≥12, <14	1,688	1.14 (1.09, 1.20)		1.15 (1.10, 1.20)		1.16 (1.12, 1.20)		1.16 (1.13, 1.19)	
<12	103	1.41 (1.16, 1.70)		1.32 (1.13, 1.55)		1.27 (1.11, 1.46)		1.30 (1.15, 1.47)	
≥14	2,782	1.17 (1.13, 1.21)		1.18 (1.14, 1.21)		1.20 (1.17, 1.23)		1.20 (1.18, 1.23)	
Bun (mg/dl)			0.1911		0.0460		0.0779		0.0369
≥9, <20	1,549	1.16 (1.08, 1.25)		1.17 (1.11, 1.24)		1.19 (1.14, 1.25)		1.20 (1.16, 1.25)	
<9	136	1.43 (1.13, 1.80)		1.48 (1.21, 1.80)		1.36 (1.18, 1.57)		1.29 (1.15, 1.44)	
≥20	3,269	1.14 (1.10, 1.17)		1.14 (1.12, 1.17)		1.16 (1.13, 1.18)		1.15 (1.13, 1.17)	
WBC (10^9^/L)			0.6638		0.4177		0.2077		0.0465
≥4, <10	1,691	1.18 (1.12, 1.23)		1.18 (1.14, 1.23)		1.19 (1.16, 1.23)		1.19 (1.15, 1.22)	
<4	90	1.11 (0.98, 1.26)		1.10 (1.00, 1.22)		1.11 (1.01, 1.20)		1.09 (1.01, 1.18)	
≥10	3,174	1.17 (1.14, 1.21)		1.18 (1.15, 1.22)		1.19 (1.16, 1.22)		1.19 (1.17, 1.22)	
RBC (10^12^/L)			0.5695		0.8637		0.7799		0.9050
≥3.5, <5.5	2,589	1.15 (1.10, 1.21)		1.17 (1.13, 1.21)		1.18 (1.14, 1.22)		1.19 (1.16, 1.23)	
<3.5	2,329	1.19 (1.15, 1.23)		1.19 (1.16, 1.22)		1.20 (1.17, 1.23)		1.19 (1.16, 1.21)	
≥5.5	37	1.23 (0.95, 1.61)		1.18 (0.92, 1.51)		1.17 (0.93, 1.48)		1.20 (0.99, 1.45)	
SOFA			0.9777		0.8875		0.5548		0.3184
<5	2,523	1.15 (1.10, 1.21)		1.16 (1.12, 1.21)		1.18 (1.14, 1.21)		1.18 (1.15, 1.21)	
≥5	2,432	1.15 (1.11, 1.19)		1.16 (1.13, 1.20)		1.18 (1.15, 1.21)		1.18 (1.16, 1.21)	
SAPSII			0.5124		0.0901		0.0195		0.0166
<39	2,511	1.16 (1.10, 1.23)		1.19 (1.14, 1.24)		1.21 (1.17, 1.25)		1.21 (1.18, 1.24)	
≥39	2,444	1.13 (1.10, 1.17)		1.14 (1.11, 1.17)		1.15 (1.12, 1.17)		1.14 (1.12, 1.17)	
EVCI			0.9266		0.1527		0.0634		0.0018
<8	2,631	1.14 (1.08, 1.20)		1.17 (1.12, 1.22)		1.19 (1.15, 1.23)		1.20 (1.17, 1.24)	
≥8	2,324	1.13 (1.10, 1.17)		1.13 (1.10, 1.16)		1.14 (1.11, 1.17)		1.13 (1.11, 1.16)	
Cardiac arrhythmias			0.3390		0.1430		0.0145		0.0300
No	3,778	1.17 (1.13, 1.21)		1.18 (1.15, 1.21)		1.20 (1.17, 1.23)		1.19 (1.17, 1.22)	
Yes	1,177	1.14 (1.09, 1.20)		1.14 (1.10, 1.19)		1.14 (1.10, 1.18)		1.14 (1.11, 1.18)	
Valvular disease			0.0406		0.0015		0.0003		<0.0001
No	4,491	1.18 (1.15, 1.22)		1.19 (1.17, 1.22)		1.20 (1.18, 1.23)		1.20 (1.18, 1.22)	
Yes	464	1.09 (1.02, 1.18)		1.08 (1.02, 1.15)		1.10 (1.04, 1.15)		1.09 (1.05, 1.14)	
Pulmonary circulation			0.4828		0.3787		0.2552		0.5388
No	4,771	1.17 (1.14, 1.20)		1.18 (1.15, 1.20)		1.19 (1.17, 1.21)		1.19 (1.17, 1.21)	
Yes	184	1.22 (1.09, 1.36)		1.23 (1.12, 1.35)		1.24 (1.14, 1.35)		1.21 (1.13, 1.31)	
Peripheral vascular			0.3585		0.5878		0.2877		0.1696
No	4,400	1.17 (1.14, 1.20)		1.18 (1.15, 1.21)		1.19 (1.17, 1.21)		1.19 (1.17, 1.21)	
Yes	555	1.22 (1.11, 1.34)		1.21 (1.11, 1.31)		1.24 (1.16, 1.32)		1.24 (1.18, 1.32)	
Hypertension			0.1739		0.1333		0.0934		0.0540
No	4,283	1.18 (1.15, 1.22)		1.19 (1.16, 1.22)		1.20 (1.17, 1.22)		1.19 (1.17, 1.21)	
Yes	672	1.12 (1.04, 1.21)		1.13 (1.06, 1.20)		1.15 (1.09, 1.21)		1.15 (1.10, 1.20)	
Chronic pulmonary			0.0274		0.0067		0.0091		0.0048
No	3,753	1.19 (1.16, 1.23)		1.20 (1.17, 1.23)		1.21 (1.18, 1.23)		1.20 (1.18, 1.22)	
Yes	1,202	1.12 (1.06, 1.18)		1.12 (1.07, 1.17)		1.14 (1.10, 1.19)		1.15 (1.11, 1.19)	
Diabetes uncomplicated			0.0186		0.2063		0.1329		0.6669
No	3,756	1.15 (1.12, 1.19)		1.17 (1.14, 1.20)		1.18 (1.16, 1.21)		1.19 (1.16, 1.21)	
Yes	1,199	1.25 (1.18, 1.32)		1.21 (1.15, 1.28)		1.23 (1.17, 1.28)		1.20 (1.16, 1.24)	
Diabetes complicated			0.7954		0.6603		0.6598		0.3443
No	4,515	1.17 (1.14, 1.21)		1.18 (1.15, 1.21)		1.19 (1.17, 1.21)		1.19 (1.17, 1.21)	
Yes	440	1.19 (1.09, 1.31)		1.21 (1.12, 1.30)		1.21 (1.14, 1.29)		1.23 (1.16, 1.30)	
Hypothyroidism			0.1152		0.1365		0.4742		0.8320
No	4,495	1.16 (1.13, 1.20)		1.17 (1.15, 1.20)		1.19 (1.17, 1.21)		1.19 (1.17, 1.21)	
Yes	460	1.24 (1.15, 1.34)		1.24 (1.16, 1.32)		1.21 (1.14, 1.29)		1.18 (1.12, 1.25)	
Renal failure			0.0887		0.0233		0.0198		0.0020
No	4,079	1.19 (1.15, 1.22)		1.19 (1.16, 1.22)		1.20 (1.17, 1.22)		1.19 (1.17, 1.22)	
Yes	876	1.12 (1.05, 1.19)		1.12 (1.06, 1.18)		1.14 (1.09, 1.19)		1.13 (1.09, 1.17)	
Liver disease			0.1413		0.1515		0.3352		0.9846
No	4,782	1.17 (1.14, 1.20)		1.18 (1.15, 1.20)		1.19 (1.17, 1.21)		1.19 (1.17, 1.21)	
Yes	173	1.30 (1.15, 1.47)		1.28 (1.15, 1.42)		1.25 (1.14, 1.37)		1.19 (1.10, 1.29)	
Coagulopathy			0.1510		0.1526		0.3453		0.0383
No	4,397	1.17 (1.14, 1.21)		1.18 (1.15, 1.21)		1.19 (1.16, 1.22)		1.19 (1.17, 1.22)	
Yes	558	1.12 (1.06, 1.19)		1.14 (1.08, 1.19)		1.16 (1.11, 1.21)		1.14 (1.10, 1.18)	
Blood loss anemia			0.5563		0.8381		0.7328		0.4609
No	4,835	1.17 (1.14, 1.20)		1.18 (1.15, 1.21)		1.19 (1.17, 1.21)		1.19 (1.17, 1.21)	
Yes	120	1.24 (1.06, 1.45)		1.17 (1.03, 1.33)		1.17 (1.05, 1.31)		1.15 (1.05, 1.26)	
Deficiency anemias			0.7989		0.0343		0.0073		0.0018
No	4,058	1.18 (1.15, 1.22)		1.20 (1.17, 1.23)		1.21 (1.19, 1.23)		1.21 (1.19, 1.23)	
Yes	897	1.17 (1.10, 1.25)		1.13 (1.07, 1.19)		1.14 (1.09, 1.19)		1.14 (1.10, 1.18)	

DBP, Diastolic blood pressure; HR, Hazard Ratio; RBC, red blood cell; PTT, partial thromboplastin time; SBP, Systolic blood pressure; WBC, white blood cell; BUN, blood urea nitrogen; PT, prothrombin time; RDW, Red Blood Cell Distribution Width; EVCI, Elixhauser-van Walraven Comorbidity Index; SAPSII, simplified acute physiology score II; SOFA, sequential organ failure assessment.

Our findings are highly dependable and consistent when evaluated both across subgroups and overall. The stratified analysis reveals a near-consensus among the results of nearly every subgroup, with effect values that are statistically significant and a 95 percent confidence interval.

The results of our subgroup analysis on diseases affecting major organs exhibit a remarkable level of uniformity and reliability. Across all variables, there is a direct association between the RDW value and the patient's likelihood of mortality in the short, medium, and long term, with higher RDW values indicating a higher risk of mortality.

## Discussion

Individuals suffering from congestive heart failure (CHF) are in the end stages of cardiac disease and have a five-year survival rate equivalent to that of malignant tumor patients (18). For the earlier identification of high-risk patients, appropriate risk stratification is critical. At present, risk stratification for heart failure patients mainly relies on clinical symptoms, imaging, and laboratory tests. The RDW is one of the components of the whole blood cell count, and it represents the degree of variation in red blood cell volume in circulating blood. Research reports have illustrated that RDW is linked to cardiovascular diseases (1, 19, 20), respiratory diseases (2), diabetes (21–23), autoimmune diseases (24), liver cancer (25), stroke (3) and so on. This article mainly introduced the relationship between RDW and CHF.

The normal erythrocyte volume is approximately 80–1,100 fL. Under some physiological conditions (pregnancy, aging, exercise, etc.) or pathological conditions (iron deficiency anemia, hemolytic anemia, etc.), erythrocyte formation is affected, resulting in an uneven erythrocyte volume. Increased RDW levels reflect impaired RBC growth (such as deficiencies in hematopoietic materials including vitamin B12, folic acid, and iron) or increased RBC breakdown (such as after hemolysis and blood transfusions), the iron deficiency and bone marrow distress that is common in heart failure could be associated with the RDW change (26). The high limit for RDW is usually 15.5% and limits greater than 15.5% are considered elevated. In clinical application, RDW combined with MCV is usually utilized for the diagnosis of anemia. For patients with reduced MCV, elevated RDW levels are considered iron deficiency anemia and globulin formation disorder anemia (27).

Any pathological changes that can affect RBC maturation, such as nutrient deficiencies (primarily iron, folic acid, and vitamin B12 deficiencies) and bone marrow suppression, may lead to elevated RDW levels, which are prevalent among heart failure patients and are linked to poor prognosis. Overactivation of the sympathetic nervous system and renin-angiotensin system among heart failure patients may also lead to elevated RDW levels and heterogeneity in RBC volume (28). The volume heterogeneity affects the oxygen-carrying capacity of RBC and further affects the scavenging of free radicals and oxidative stress response (29). In addition, many inflammatory markers associated with heart failure, including the erythrocyte sedimentation rate, hypersensitive C-reactive protein level, or WBC count, are strongly associated with RDW (30).

Elevated RDW levels may lead to tissue hypoperfusion. Several research reports have illustrated that RDW levels are positively linked to central venous pressure and negatively correlated with mixed venous oxygen saturation (31). Low erythropoietin production and decreased serum albumin may be related to the mechanism by which RDW affects the prognoses of heart failure patients.

RDW is economical, quick, and simple to detect and can be combined with other prognostic indicators for more specific risk stratification and early treatment of these patients (32–34).

Multiple research reports have evaluated the link between RDW and the clinical outcomes of cardiovascular events. Remo et al. showed that RDW is a robust indication of poorer long-term outcomes among acute heart failure patients, and its predictive significance is superior compared to other well-established biological markers or risk factors (35). Patients exhibiting greater RDW levels were found to have greater higher Charlson Index scores and more comorbid conditions. This is consistent with our research. Our study showed that high RDW levels correspond to high EVCI scores. Yan Borne et al. illustrated that RDW was linked to the long-term prevalence of first hospital admission in middle-aged individuals with HF (36). Domingo et al. pointed out that elevated RDW level upon discharge was linked to unfavorable long-term outcomes, irrespective of anemia status and levels of hemoglobin (37). Andras et al. found that enhanced RDW levels allowed for an accurate prediction of the long-term death of cardiac resynchronization therapy patients independent of NT-proBNP. RDW enhances risk stratification and might promote accurate patient identification for cardiac resynchronization treatment, according to a reclassification study (38). Muhlestein et al. suggested that greater initial RDW levels during hospitalization for HF were linked to 30-day all-cause readmission, longer length of stay, and 30-day mortality, implying that early-stage RDW levels could assist in personalized treatment and prognosis improvement (39). G. Michael et al. pointed out that RWD was a significant independent biomarker of morbidity and death in two large contemporary heart failure populations. Determining how and why this biomarker is linked to outcomes might reveal new information about the pathophysiology of heart failure (40).

Our findings demonstrated a positive link between RDW and 30-day, 90-day, 365-day, and 4-year all-cause mortality among CHF patients receiving intensive care after adjustment for other covariates. RDW levels had a nonlinear relationship with 30-day, 90-day, 365-day, and 4-year all-cause mortality in CHF patients in critical condition, presenting a U-shaped curve. The greatest benefit was observed in the first 30 days and this benefit was reduced at 90 days, 365 days, and 4 years. Subgroup analysis helped us better understand trends in all-cause mortality and RDW levels in different populations. In a subgroup analysis, we found that RDW levels had less interaction with 30-day, 90-day, 365-day, and 4-year all-cause death in CHF patients in critical condition, and the findings were reliable and stable. The K-M survival curve confirmed our hypothesis that RDW levels were positively correlated with long-, medium-, and short-term all-cause mortality.

The clinical implications of this research include the following: (1) we observed curved relationships between RDW levels and 30-day, 90-day, 365-day, and 4-year all-cause death among CHF patients receiving intensive care; (2) the findings obtained from this research can aid the establishment of diagnostic and prognostic RDW models for CHF patients in the short, medium and long term.

Our study has some advantages. (1) The Mimic-III database is a comprehensive publicly accessible repository with reliable data, numerous covariables, and sufficient adjustment for confounding factors. (2) Our investigation uncovered a significant non-linear association between the red blood cell distribution width and CHF patients, which may has ramifications for the use of illness markers in the future to help with mortality prediction. (3) This study was a real-world study without invasive damage to patients. (4) The independent variables of interest were presented as categorical and continuous variables. This strategy has the potential to decrease data analysis contingencies and improve the outcomes' robustness. (5) The interaction test results obtained for different subgroups in this study could better enable us to conduct data analysis and draw stable conclusions.

This research has some drawbacks as follows: (1) the research object of this study was CHF, so there are some limitations in the generality and extrapolation of this study, and it is not applicable to other patients. (2) The RDW value was recorded for the first time upon patient admission to the ICU without observation of laboratory follow-up data, so the results may be biased. (3) There may be some potential confounding factors that were not included in the laboratory examination on the first day following ICU admission, so our results may also be affected by other confounding factors. For example, NTproBNP and C-reactive protein plasma levels are more powerful prognostic predictors in heart failure patients. However, the data obtained at baseline were largely missing. (4) Erythrocyte transfusion is an important potential confounding factor. However, there was no record of erythrocyte transfusion prior to admission to the hospital or ICU in the Mimic-III V. 1.4 database. (5) Our research was unable to elucidate the underlying mechanisms of RDW and all-cause mortality, which requires further study. (6) We were unable to obtain serum iron ion data for ICU patients, which is an important confounding factor that may have affected our results. We will consider this confounding factor in future studies, highlighting the possible impact of unmeasured factors (e.g., iron deficiency) on our conclusions, in order to improve the design of our study.

## Conclusion

Our investigation uncovered a significant non-linear association between the red blood cell distribution width and CHF patients. The RDW levels had a u-shaped connection with 30-day mortality. The RDW level was associated with an elevated risk of long-, medium-, and short-term all-cause death among CHF patients. In conclusion, RDW is an objective marker for determining the severity of HF and anticipating the mortality risk in CHF patients, and it is a convenient, inexpensive, and easy-to-obtain effective indicator.

## Data Availability

Publicly available datasets were analyzed in this study. This data can be found here: The datasets used in this investigation may be found in the MIMIC-III database (https://mimic.mit.edu/).
